# Understanding cancer disease status and what it means for intensivists

**DOI:** 10.1007/s00063-025-01320-6

**Published:** 2025-08-29

**Authors:** Nina Buchtele, Laveena Munshi

**Affiliations:** 1 Intensive Care Unit 13i2, Department of Medicine I, Medical University of Vienna, Währinger Gürtel 18–20, 1090 Vienna, Austria; 2 Interdepartmental Division of Critical Care Medicine, Mount Sinai Hospital, Sinai Health System, University of Toronto, Toronto, Canada

**Keywords:** Critical care oncology, Treatment outcome, Prognosis, Interdisciplinary care, Advance care planning, Intensivmedizinische Onkologie, Behandlungsergebnis, Prognose, Interdisziplinäre Versorgung, Advance Care Planning

## Abstract

A thorough understanding of oncological disease status is crucial for managing critically ill patients with cancer. The cancer trajectory predisposes patients to the type of critical illness they could develop and shapes the likelihood of reversibility and the chance for meaningful recovery, including continuation of therapy. This review outlines how disease status—whether new diagnosis, remission, stable disease, or progression—directly impacts differential diagnosis and treatment goals in the intensive care unit (ICU). Prognosis can be subdivided into (1) the comorbid cancer condition and (2) the acute critical care condition. Factors that impact prognosis may be similar including patient frailty, extent of organ failure, and tumor-related factors. While ICU survival remains an important patient-centered outcome, long-term outcomes such as return to treatment and acceptable quality of life have become increasingly important as ICU survival has improved over the past decades. Clear communication about patient values helps align care with realistic goals and avoid disproportionate ICU treatment. However, a critical element of establishing realistic goals includes a thorough understanding of the oncologic disease status. Close collaboration between intensivists and oncologists improves prognostication, treatment planning, and advance care discussions. Early recognition of high-risk patients and clear escalation or limitation pathways help ensure timely ICU transfer when needed. After ICU discharge, coordinated follow-up and rehabilitation support recovery and candidacy for further oncological treatment. An integrated, goal-directed approach enables tailored care for this complex patient group and supports shared decision-making throughout the continuum of cancer care.

## Abstract (de)

Ein tiefes Verständnis für das onkologische Krankheitsstadium ist entscheidend für die Behandlung kritisch kranker Patienten mit Krebs. Der Verlauf der Krebserkrankung prädisponiert Patienten für die Art kritischer Erkrankung, die bei ihnen entstehen könnte, und prägt die Wahrscheinlichkeit von Reversibilität und die Chance auf eine nennenswerte Erholung, einschließlich Fortsetzung der Therapie. In der vorliegenden Übersichtsarbeit wird skizziert, wie das Krankheitsstadium – ob Erstdiagnose, Remission, stabile Erkrankung oder Progression – direkten Einfluss auf die Differenzialdiagnose und Therapieziele auf der Intensivstation (ICU) hat. Die Prognose kann unterteilt werden in (1) die als Komorbidität bestehende Krebserkrankung und (2) den akuten kritischen Zustand. Die Faktoren mit Einfluss auf die Prognose können ähnlich sein, einschließlich Gebrechlichkeit des Patienten, Ausmaß des Organversagens und tumorabhängiger Faktoren. Während das Überleben auf der ICU ein wichtiges patientenzentriertes Outcome bleibt, ist es maßgeblich, dass Langzeitergebnisse wie die Wiederaufnahme der Therapie und eine akzeptable Lebensqualität zunehmend wichtiger geworden sind, das die Überlebensrate auf der ICU über die vergangenen Jahrzehnte besser geworden ist. Klare Kommunikation über die Werte des Patienten tragen dazu bei, die Versorgung mit realistischen Zielen in Einklang zu bringen und unangemessene Therapien auf der ICU zu vermeiden. Allerdings gehört zur Setzung realistischer Ziele als wesentliches Element ein tiefes Verständnis des onkologischen Krankheitsstadiums. Enge Zusammenarbeit zwischen Intensivmedizinern und Onkologen verbessert die prognostischen Voraussagen, Therapieplanung und Vorsorgegespräche. Die Früherkennung von Hochrisikopatienten und klare Pfade für Ausweitung oder Begrenzung von Versorgung dienen der Sicherstellung einer rechtzeitigen Verlegung auf die ICU im Bedarfsfall. Nach Entlassung von der ICU wird die Erholung und die mögliche Eignung für weitere onkologische Behandlung durch eine koordinierte Nachsorge und Rehabilitation unterstützt. Ein integrierter, zielgerichteter Ansatz ermöglicht eine angepasste Versorgung dieser komplexen Patientengruppe und unterstützt die gemeinsame Entscheidungsfindung während der gesamten Krebsbehandlung.

## Introduction

Improved intensive care unit (ICU) mortality in patients with cancer is a major paradigm shift in critical care outcomes of this vulnerable population. Given this, we are now at an age where we can turn our attention to longer term outcomes of this population. Importantly, survival with a good quality of life is often only one objective at the time point of ICU admission. Returning to a clinical state where they can resume ongoing cancer-curative or life-prolonging treatment after critical illness survival is just as important for many. Given this, a thorough understanding of not just the acute care condition but also the oncological disease status of patients is crucial.

The trajectory of cancer influences both the likelihood of a critical illness being reversible and the potential for meaningful recovery, including the continuation of cancer therapy. Intensivists play a vital role in this clinical landscape, balancing acute care with a long-term oncologic objective.

This review highlights a key aspect of oncologic critical care: the cancer disease status and how it should be used to guide clinical decision making in critically ill patients. In this context this review aims to provide an overview for intensivists on relevant oncological factors, such as definitions of disease status, how treatment goals should be tailored, and how interprofessional collaboration between oncologists and intensivists are necessary and improve outcome.

## Prognostic factors in critically ill cancer patients

**Fig. 1:**
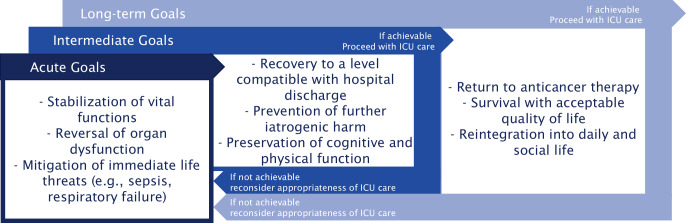
Treatment goals for critically ill cancer patients

Outcomes across critically ill patients with cancer is predominantly determined by patient-related, and tumor-related factors. Although outcome has improved over the past decades, it is imperative that realistic treatment goals should be discussed with the patient taking into consideration oncologic and critical care aspects. These goals will include acute care, intermediary care, and long-term care goals. This is accomplished only through interdisciplinary conversations between the oncology and ICU teams (Fig. 1).

An important consideration for ICU survival is reversibility of organ failure. However, this may be challenging to prognosticate at the time point of ICU admission. Given this, the concept of time-limited trials have been created—which is a trial of ICU care for a fixed amount of time with specific goals established [2]. The success of this concept relies on interdisciplinarity, shared-decision making, and regular meetings throughout critical illness to ensure appropriateness of intensive care treatment. The optimal duration of time-limited trials cannot be generalized, but may be longer for patients with hematological disease than for patients with solid tumors [3].

**Fig. 2:**
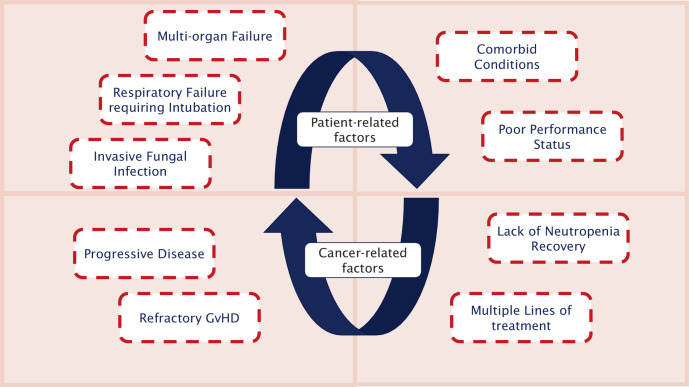
Patient- and cancer-related factors associated with poor outcome. *GvHD* graft vs. host disease

Besides organ-failure reversibility, several further factors need to be considered for individual prognosis. For patients fulfilling several poor prognostic criteria (Fig. 2), treatment goals should focus on symptom control and palliation.

### Short- and long-term outcomes: focus on return to treatment

Given improved outcomes in modern ICU and oncologic care, outcome measurements have shifted beyond ICU survival to longer-term survival and quality of life. In this context, long-term functional outcome has become an important outcome to be considered [5, 6]. In critically ill patients with cancer, this is a particularly important outcome given that it is often a prerequisite for return to cancer care [5, 7]. For the intensivist, it is of utmost importance to understand which state needs to be achieved for ongoing cancer therapy [8]. A previously young and healthy patient with new diagnosis of acute myeloid leukemia may have fair chance of a cure of their disease. If they develop critical illness, it is necessary to understand what state of recovery they would need to achieve for ongoing curative treatment (intensive chemotherapy or allogeneic stem cell transplant). This may include both acceptable organ function and a moderate-to-high level of fitness. Knowledge of these outcomes may direct prognostic conversations at the outset or during the ICU stay.

On the other end of the spectrum, certain advanced cancer subtypes have become more of chronic diseases with acceptable quality of life in the age of novel therapies for metastatic cancers [9]. However, the majority of patients with advanced stage cancer require continuity of maintenance therapy, e.g., checkpoint inhibitors or other targeted therapies, for adequate cancer control. Permanent organ dysfunction after ICU stays needs to be considered as an important barrier that could disqualify from the continuation of cancer therapy. Again, several therapies require a distinct performance status to be administered (e.g., chemotherapy, surgery), but others exist, which can be applied in more frail patients (e.g., radiation) [8]. Knowledge of criteria for candidacy of ongoing treatment may inform the extent of ICU management if these patients develop critical illness.

## Integrating disease status and treatment potential in ICU decision-making

Disease status typically falls into several categories, each with specific implications for intensive care management. Disease status is determined after predefined timeframes during or after completion of cancer treatment. Understanding disease status is essential to inform whether escalation to ICU care should or should not be offered, whether it may help in developing a differential diagnosis of the acute illness, and whether it has important implications on realistic long-term outcomes, especially in the light of return to treatment to effectively control malignant disease [10].

### New diagnosis

A cancer diagnosis established during or shortly before ICU admission often represents the tip of an aggressive clinical iceberg. These patients may present with disease-related complications such as tumor lysis syndrome, leukostasis, spinal cord compression, or malignant effusions [11, 12]. For the intensivist, it is vital to recognize that the critical illness is likely driven by the underlying, untreated malignancy. Aggressive diagnostic and supportive interventions are justified if the cancer is potentially curable or significantly controllable and the patient has other favorable ICU prognostic factors (e.g., low frailty, few additional comorbidities). Occasionally a patient with newly diagnosed cancer will present with concomitant severe infection leading to septic shock or pneumonia prior to cancer treatment.

**Fig. 3:**
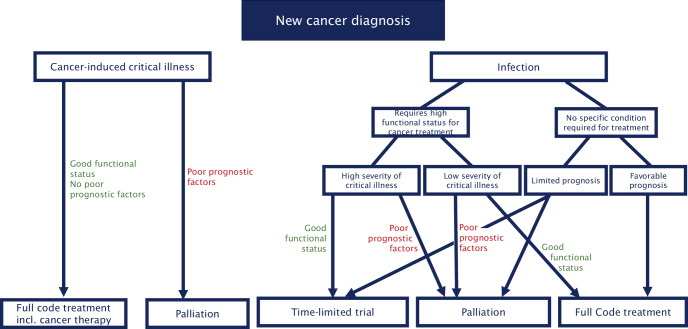
Decision tree for assessing treatment goals in patients with new cancer diagnosis. *incl*. including

Escalation to ICU care should consider whether anti-infectives and anti-cancer therapy could be administered concurrently (if the two are related such as a postobstructive pneumonia in the setting of lung cancer), or if cancer therapy should be delayed. In the latter situation, it is important to understand what state the patient would need to be in to receive cancer therapy and whether achieving that state is realistic. For example, if a patient newly developed pneumonia and severe acute respiratory distress syndrome but also has been diagnosed with metastatic ovarian cancer, one would need to understand from the oncologist what treatment options are available and the functional state the patient would need to be in to be eligible. If the patient would need to independently walk into an outpatient clinic to be a candidate for systemic treatment, then they may only choose a time-limited trial of ICU understanding that if their ICU stay is prolonged beyond a few weeks of invasive mechanical ventilation, the likelihood of timely recovery to be functionally independent to receive systemic cancer treatment would be low (Fig. 3).

### Remission

Remission means a decrease in or disappearance of signs and symptoms of cancer, which is determined after any therapy, except for palliative treatment. With complete remission, there may exist residual cancer but it is not detected. We distinguish between complete remission, where all signs and symptoms of cancer have disappeared, and partial remission, where some, but not all signs and symptoms have disappeared. Partial remission may also be called partial response. Depending on the cancer entity, various diagnostic criteria apply for the definition of remission.

Patients in complete or partial remission are much less likely to develop new onset organ dysfunction due to their underlying malignancy (e.g., leukemic pulmonary infiltration or airway obstruction due to tumor mass). However, they may have treatment-induced causes of critical illness whether it be due to treatment toxicity or immunosuppression-induced sepsis [11].

Infections are common complications and may include typical bacterial or viral pathogens as well as opportunistic, fungal, or atypical organisms. Ongoing immunosuppressive therapies—such as postallogeneic stem cell transplantation regimens or maintenance targeted therapies in solid tumors—can significantly increase this risk depending upon the mechanism of immunosuppression. High-risk patients should receive prophylaxis against *Pneumocystis jirovecii* and/or invasive fungal infections; however, adherence and factors impairing drug efficacy (e.g., diarrhea) must be carefully assessed. In remission, late toxicities from prior cancer therapies must also be considered. Cumulative toxicity increases the risk of long-term organ dysfunction.

These include but are not limited to:

- Cardiotoxicity from anthracyclines (e.g., doxorubicin, daunorubicin) used in breast cancer, lymphoma, or leukemia.
- Neurotoxicity from methotrexate, especially in acute lymphoblastic leukemia (ALL) or central nervous system (CNS) lymphoma.
- Pulmonary toxicity from cisplatin, commonly used in lung cancer.

When determining goals of care for patients in remission, the reversibility of organ failure and the potential for long-term quality of life are key considerations—alongside functional status. An ICU trial of individual duration may be appropriate for most, provided meaningful recovery is achievable. In patients with complete remission, excellent functional status, and end-stage organ toxicity from prior treatment, transplant candidacy should be explored when the risk of cancer recurrence is low.

### Stable disease

Cancer that is neither decreasing nor increasing in extent or severity is considered stable disease. Patients with stable cancer disease may rarely present with cancer-related critical illness. Still, attention must be paid to very sensitive organs. An example would be new seizures in patients with brain cancer or cerebral metastasis.

In critically ill patients with stable disease, it is essential to assess how disease stability was achieved, whether maintenance therapy is ongoing or planned, and whether curative treatment options remain. Functional status and oncologic prognosis—particularly the ability to tolerate further therapy—are key factors in shaping goals of care. Poor prognostic indicators should be considered equally but patients with reversible organ failure should be offered ICU care.

### Progression

Progressive disease reflects cancer growth. Intensivists must thoroughly ascertain whether the critical illness is a direct consequence of tumor growth, such as airway obstruction, intracranial pressure elevation, or metastatic complications. However, superimposed conditions, such as infections or metabolic imbalances need to be considered. It is imperative to understand whether additional alternative treatment options are available to address disease progression.

**Fig. 4:**
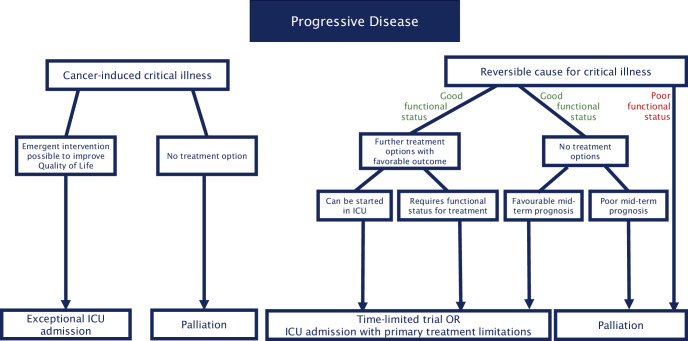
Decision tree for assessing treatment goals in patients with progressive cancer. *ICU* intensive care unit

Progressive disease is generally associated with poor ICU outcomes, but selected patients may still benefit from ICU care, e.g., those with septic shock from a treatable source requiring brief vasopressor support. Full-code treatment should be reserved for highly selected cases—particularly if effective alternative cancer treatment options are available; however, ICU admission with predefined limitations (e.g., no invasive ventilation) or an ICU trial can be considered and discussed (Fig. 4).

### Undetermined disease status

Patients with undetermined disease status—i.e., those recently treated for cancer but not yet restaged—commonly present with critical illness related to treatment toxicity. This includes infections due to immunosuppression or neutropenia, bleeding from thrombocytopenia or coagulopathy, cardiogenic shock from chemotherapy-induced cardiotoxicity, or pneumonitis from checkpoint inhibitors. The type of treatment, timing, and degree of immunosuppression help guide the differential diagnosis.

**Fig. 5:**
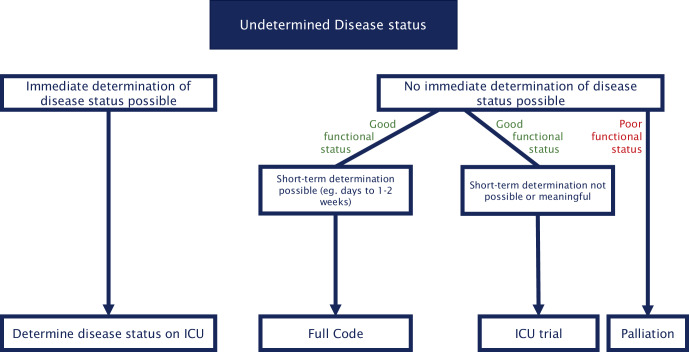
Decision tree for assessing treatment goals in patients with undetermined disease status. *ICU* intensive care unit

These cases pose a particular challenge for intensivists in defining goals of care. Close collaboration with oncology is essential to determine when and how disease status can be reassessed. Fig. 5 gives a suggestion of how goals of care should be addressed in patients with undetermined disease status.

## Interprofessional roles in the ICU and beyond

Effective care requires seamless integration between hematology/oncology and intensive care teams during the entire treatment of cancer patients.

### Advance care planning in the outpatient setting

One in seven patients with newly diagnosed hematologic malignancy requires unscheduled admission to an ICU within one year of diagnosis [13]. Five percent of patients with solid cancer require ICU admission within 2 years of diagnosis [14]. Advance directives, treatment limitations, and preferences should be documented, especially for patients with advanced or high-risk disease [15]. These aspects should be discussed as early as possible in the course of the disease; however, several challenges exist [15]. In the early phase of cancer, advance care planning about possible ICU admission may be difficult because prognosis is unclear, patients may be overwhelmed, information remains incomplete, and the concept of limiting care can feel incompatible with the patient’s and relatives’ optimism surrounding treatment. Good communication and also revisiting advance care planning over time are key to making these discussions meaningful and acceptable [16]. It is also important to highlight how these conversations are dynamic and the recommendations from the medical team may change as the patient’s disease status evolves.

### Identifying high-risk patients on the ward

Intensivists can help oncologists by providing risk awareness, clear escalation pathways, practical training, joint rounds, and (continuation of) advance early planning—all of which improve early recognition and timely ICU referral for high-risk cancer patients. While this approach and collaboration remain ideal, the reality is that resource shortages often limit clinical practice. It may not be feasible for intensivists for meet with all high-risk patients; therefore, having a system on how to identify potentially deteriorating patients may help provide a structure for on-demand visits by intensivists, especially if ICU capacities limit early admission. In general, as soon as any sign of new organ dysfunction is evident, a potential benefit of ICU admission should be discussed.

**Fig. 6:**
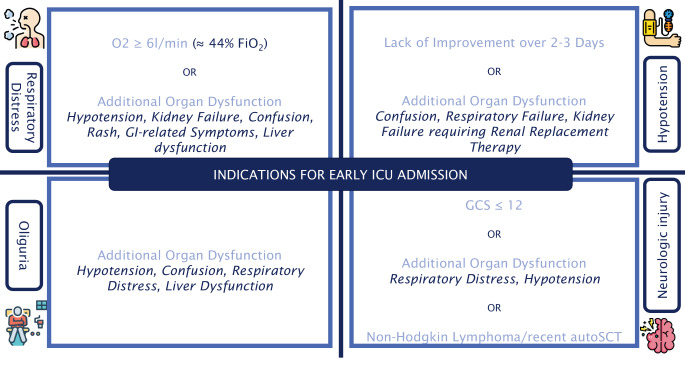
Indications for early intensive care unit (ICU) admission in cancer patients. *GI* gastrointestinal, *autoSCT* autologous stem cell transplant, *FiO2* fraction of inspired oxygen, *GCS* Glasgow Coma Scale

Patients with high-risk acute myeloid leukemia (e.g., elevated white blood cell count, high-risk subtype, or disseminated intravascular coagulation) combined with early clinical symptoms such as respiratory distress, tachycardia, or hypotension should be considered for ICU admission [12, 17]. Similarly, patients with aggressive malignancies and high tumor burden who have a clinical tumor lysis syndrome risk score above 10 (based on elevated LDH, phosphate, and disseminated intravascular coagulation) may benefit from proactive ICU transfer to prevent severe complications [18]. For other cancer patients, criteria depicted in Fig. 6 may guide identification of patients who may benefit from timely transfer to intensive care to prevent further deterioration [19–22].

### The hematologist and oncologist in the ICU

Close collaboration with the oncologist during the patient’s ICU stay is a key component of management and has shown to improve survival [23]. The following key points should be provided by the oncologist on admission and throughout the ICU stay to frame and adapt treatment goals appropriately:

- Patient’s functional status and preferences
  - Current performance status (e.g., Eastern Cooperative Oncology Group [ECOG] or Clinical Frailty Scale)
  - Patient’s values and preferences for any treatment limitations
- Disease trajectory
  - Current disease status and associated cancer prognosis
  - Recent treatments administered and response
- Future cancer therapy
  - Type and timing of treatment and patient’s required condition (functional status, organ function)
  - Alternative treatment options

### After successful discharge from the ICU

After ICU discharge, patients often require specialized care to minimize post-intensive care syndrome [24]. Important aspects include management of delirium, addressing loss of independence and any psychiatric conditions such as anxiety, depression or posttraumatic stress disorder that may develop after ICU. Therapies, including physiotherapy with adequate nutritional support, ergotherapy, and psychological support should continue on regular wards. Social workers should be incorporated into the care team to help with potentially newly established frailty to prepare patients’ readiness for discharge home or help in organizing care facilities. Likewise, intensivists should communicate any discussion about treatment limitations and whether another ICU stay, in case of worsening function would be advisable. Additionally, intensivists should give a recommendation on the type and frequency of monitoring (vital signs and laboratory tests) required for the first few days after transfer. Most importantly, open dialogue and clear coordination help ensure that both nursing and medical tasks are aligned to meet the complex needs of prior critically ill patients.

## Practical conclusion

Caring for critically ill cancer patients requires intensivists to understand the patient’s disease status, prognosis, and treatment needs. Whether cancer is newly diagnosed, in remission, stable, or progressive determines the likely cause of acute illness and shapes goals of care. Prognosis depends on patient frailty, organ failures, disease status, and treatment options. While intensive care unit (ICU) survival is a clear outcome, quality of life and the chance to continue cancer therapy are more meaningful. Intensivists must weigh what can be done against what should be done to avoid disproportionate treatment. Close collaboration with oncologists helps define prognosis, treatment goals, and care limitations. Early identification of high-risk patients and clear escalation plans enable timely ICU admission. After ICU discharge, coordinated follow-up supports recovery and readiness to resume cancer therapy.

## Declarations

For this article no studies with human participants or animals were performed by any of the authors. All studies mentioned were in accordance with the ethical standards indicated in each case.
